# APOE e4 Genotype Predicts Severe COVID-19 in the UK Biobank Community Cohort

**DOI:** 10.1093/gerona/glaa131

**Published:** 2020-05-26

**Authors:** Chia-Ling Kuo, Luke C Pilling, Janice L Atkins, Jane A H Masoli, João Delgado, George A Kuchel, David Melzer

**Affiliations:** 1 Connecticut Convergence Institute for Translation in Regenerative Engineering, University of Connecticut Health, Farmington; 2 Center on Aging, University of Connecticut Health, Farmington; 3 Epidemiology and Public Health Group, University of Exeter Medical School, UK; 4 Department of Healthcare for Older People, Royal Devon and Exeter Hospital, UK

**Keywords:** COVID-19, APOE, Genetic

The novel respiratory disease COVID-19 produces varying symptoms, with fever, cough, and shortness of breath being common. In older adults, we found that preexisting dementia is a major risk factor (odds ratio [OR] = 3.07, 95% CI: 1.71 to 5.50) for COVID-19 severity in the UK Biobank (UKB) (1). In another UK study of 16,749 patients hospitalized for COVID-19 (2), dementia was among the common comorbidities and was associated with higher mortality. Additionally, impaired consciousness, including delirium, is common in severe cases (3). The *ApoE* e4 genotype is associated with both dementia and delirium (4), with the e4e4 (homozygous) genotype associated with a 14-fold increase in risk of Alzheimer’s disease (5) compared to the common e3e3 genotype, in populations with European ancestries. We, therefore, aimed to test associations between *ApoE* e4 alleles and COVID-19 severity, using the UKB data.

UKB is a community cohort currently aged 48 to 86 (6). COVID-19 laboratory test results for UKB participants in England are available from March 16 to April 26, 2020, the peak period of COVID-19 incidence in the current outbreak. During this period, COVID-19 testing was largely restricted to hospital inpatients with clinical signs of infection, and therefore test positivity is a marker of severe COVID-19 infection (7).

We analyzed UKB data from genetically European ancestry participants (8) (*n* = 451,367, 90% of sample) attending baseline assessment centers in England (*n* = 398,073), excluding participants who died before the epidemic (*n* = 15,885). Single nucleotide polymorphism (SNP) data for rs429358 and rs7412 was used to determine *ApoE* genotypes: *ApoE* e4e4 homozygotes (*n* = 9,022, 3%), e3e4 (*n* = 90,469, 28%), and e3e3 (most common genotype, *n* = 223,457, 69%) genotype groups (final *n* = 322,948). Mean age was 68 years (*SD* = 8) with 176,951 females (55%). There were 622 positive COVID-19 patients (Table 1) including 37 with e4e4 (positivity rate: 410/100,000) and 401 with e3e3 (179 per 100,000). A logistic regression model was used to compare e3e4 or e4e4 genotypes to e3e3 for COVID-19 positivity status, adjusted for: sex; age at the COVID-19 test or age on April 26, 2020 (the last test date); baseline UKB assessment center in England; genotyping array type; and the top five genetic principal components (accounting for possible population admixture).

**Table 1.** Risk of Severe COVID-19, Comparing Participants With *ApoE* e3e4 or e4e4 to e3e3 Genotypes in UK Biobank

	*n*	Negative or not Tested	Positive	Positivity Rate per 100,000	OR (95% CI)^a^	*p*-value
All						
e3e3	223,457	223,056	401	179	-	-
e3e4	90,469	90,285	184	203	1.14 (0.95, 1.35)	.15
e4e4	9,022	8,985	37	410	2.31 (1.65, 3.24)	1.19E-06
Excluding dementia						
e3e3	222,968	222,574	394	177	-	-
e3e4	90,013	89,840	173	192	1.09 (0.91, 1.31)	.338
e4e4	8,877	8,840	37	417	2.39 (1.71, 3.35)	4.26E-07
Excluding hypertension						
e3e3	151,018	150,792	226	150	-	-
e3e4	61,249	61,157	92	150	1.00 (0.79, 1.28)	.981
e4e4	6,120	6,098	22	359	2.41 (1.56, 3.74)	8.21E-05
Excluding coronary artery disease						
e3e3	204,017	203,684	333	163	-	-
e3e4	82,099	81,948	151	184	1.13 (0.93, 1.37)	0.207
e4e4	8,164	8,132	32	392	2.43 (1.69, 3.50)	1.65E-06
Excluding type 2 diabetes						
e3e3	211,482	211,136	346	164	-	-
e3e4	85,983	85,827	156	181	1.11 (0.92, 1.34)	.275
e4e4	8,616	8,581	35	406	2.51 (1.77, 3.55)	2.42E-07

*Note*: ^a^Adjusted for sex, age at the COVID-19 test or age on April 26, 2020 (the last test date), assessment center in England, genotyping array type, and the top five genetic principal components.

*ApoE* e4e4 homozygotes were more likely to be COVID-19 test positives (OR = 2.31, 95% CI: 1.65 to 3.24, *p* = 1.19 × 10^–6^) compared to e3e3 homozygotes (Table 1). The association was similar after removing participants with *ApoE* e4 associated diseases that were also linked to COVID-19 severity: participants without dementia (OR = 2.39, 95% CI: 1.71 to 3.35); hypertension (OR = 2.41, 95% CI: 1.56 to 3.74); coronary artery disease (myocardial infarction or angina) (OR = 2.43, 95% CI: 1.69 to 3.50) or type 2 diabetes (OR = 2.51, 95% CI: 1.77 to 3.55) (Table 1), based on preexisting diagnoses from baseline self-reports or hospital discharge statistics (updated to March 2017). The estimates were little changed using 136,146 participants with additional general practice data (up to 2017): participants without dementia (OR = 2.53, 95% CI: 1.46 to 4.39); hypertension (OR = 2.67, 95% CI: 1.34 to 5.32); coronary artery disease (OR = 2.86, 95% CI: 1.65 to 4.98) or type 2 diabetes (OR = 2.73, 95% CI: 1.57 to 4.76). The results were also similar after excluding 51,430 participants related to the third degree or closer (OR = 2.34, 95% CI: 1.62 to 3.38). Of 622 included participants who tested positive for COVID-19, 417 (67%) were noted to the laboratory to be inpatients when the sample was taken: unfortunately, data on later admission to hospital is not available (7). Including only those known to have been inpatients when tested made little difference to the excess risk associated with *ApoE* e4e4 status (OR = 2.32, 95% CI: 1.54 to 3.29), compared to OR = 2.31 (95% CI: 1.65 to 3.24) using all the tested samples.

In conclusion, the *ApoE* e4e4 allele increases risks of severe COVID-19 infection, independent of preexisting dementia, cardiovascular disease, and type-2 diabetes. *ApoE* e4 not only affects lipoprotein function (and subsequent cardio-metabolic diseases) but also moderates macrophage pro-/anti-inflammatory phenotypes (9). The novel coronavirus SARS-CoV-2 causing COVID-19 uses the ACE2 receptor for cell entry. ACE2 is highly expressed in type II alveolar cells in the lungs, where *ApoE* is one of the highly co-expressed genes (10). Further investigation is needed to understand the biological mechanisms linking *ApoE* genotypes to COVID-19 severity.
